# Attackenangst bei Migräne: Diagnostik und Behandlung

**DOI:** 10.1007/s00482-023-00711-y

**Published:** 2023-04-18

**Authors:** Timo Klan, Anke Diezemann-Prößdorf, Anna-Lena Guth, Charly Gaul, Michael Witthöft

**Affiliations:** 1 Psychologisches Institut, Johannes Gutenberg-Universität Mainz, Wallstr. 3, 55099 Mainz, Deutschland; 2 DRK Schmerz-Zentrum Mainz, Mainz, Deutschland; 3 Kopfschmerzzentrum Frankfurt, Frankfurt, Deutschland

**Keywords:** Kopfschmerz, Fear-avoidance, Angst, Verhaltenstherapie, Fragebogen, Headache, Fear-avoidance, Anxiety, Behavioral therapy, Questionnaire

## Abstract

Im Kontext von Kopfschmerzerkrankungen beschreibt der Begriff „Attackenangst“ die Furcht vor dem Auftreten einer Kopfschmerzattacke. Ein Übermaß an Attackenangst kann sich ungünstig auf den Krankheitsverlauf bei Migräne auswirken und zu einer Verstärkung der Migräneaktivität führen. Zur Diagnostik von Attackenangst stehen der kategoriale Ansatz (Attackenangst als spezifische Phobie) sowie der dimensionale Ansatz (Erfassung des Angstausmaßes mittels Selbstbeurteilungsfragebogen) zur Verfügung. Als Selbstbeurteilungsfragebogen liegt mit dem 29 Items umfassenden *Fragebogen zur Attackenangst bei Migräne* (FAMI) ein ökonomisches Messinstrument mit guten psychometrischen Eigenschaften vor. Zur Behandlung von Attackenangst bei Migräne existieren neben medikamentösen Maßnahmen auch gezielte verhaltenstherapeutische Interventionen. Die nebenwirkungsarmen verhaltenstherapeutischen Interventionen sind an die Behandlung von gängigen Angststörungen (z. B. Agoraphobie) angelehnt. Ein Wirksamkeitsnachweis für spezifische Interventionen zur Behandlung von Attackenangst bei Migräne steht noch aus. Im Rahmen der Kopfschmerzanamnese und -behandlung sollte Attackenangst dennoch adressiert werden.

**Zusatzmaterial online:**

Die Online-Version dieses Beitrags (10.1007/s00482-023-00711-y) enthält den Fragebogen zur Attackenangst bei Migräne (FAMI).

## Abstract (en)

In the field of headache disorders, the term “fear of attacks” refers to the fear of a headache attack occurring. Excessive fear of attacks may worsen the course of a migraine and lead to an increase in migraine activity. In the assessment of attack-related fear, a categorical (fear of attacks as a specific phobia) and a dimensional approach (measuring the extent of fear using a questionnaire) are available. The 29-item Fear of Attacks in Migraine Inventory (FAMI) is an economic self-report questionnaire for the assessment of attack-related fear, and it has good psychometric properties. The treatment of attack-related fear includes behavioral interventions as well as pharmacological therapy. Behavioral interventions have few side effects and are based on the treatment of common anxiety disorders (e.g., agoraphobia). Although the evidence of existing treatments is sparse, attack-related fear should be considered in routine care.

Angst kann zur Chronifizierung von Schmerz beitragen, was durch das Fear-avoidance-Modell gut erklärt werden kann. Auch bei Kopfschmerzerkrankungen kann sich Angst ungünstig auf den Krankheitsverlauf auswirken. Eine spezifische Form der Angst im Kontext einer Migräneerkrankung ist die Angst vor dem Auftreten einer Migräneattacke („Attackenangst“). Nachfolgend werden diagnostische Verfahren einschließlich eines Selbstbeurteilungsfragebogens zur Erfassung von Attackenangst bei Migräne sowie entsprechende Behandlungsoptionen vorgestellt.

## Einleitung

Migräne ist eine primäre Kopfschmerzerkrankung von hoher Prävalenz. Weltweit sind 13,8 % der Frauen und 6,9 % der Männer betroffen [41]. Die Erkrankung kann zu hohen Einschränkungen der Lebensqualität führen und ist unter den Top Ten der Ursachen für Beeinträchtigung [39]. Kennzeichen der Migräne sind wiederkehrende Kopfschmerzattacken von mittlerer bis hoher Intensität mit einer Dauer von 4 bis 72 h [14]. Begleitsymptome der Attacke sind Übelkeit sowie Licht- und Geräuschempfindlichkeit. Bei etwa 30 % der Migränebetroffenen treten vor der Kopfschmerzphase Aurasymptome (neurologische Reiz- oder Ausfallerscheinungen) auf [23].

**Figure 1:**
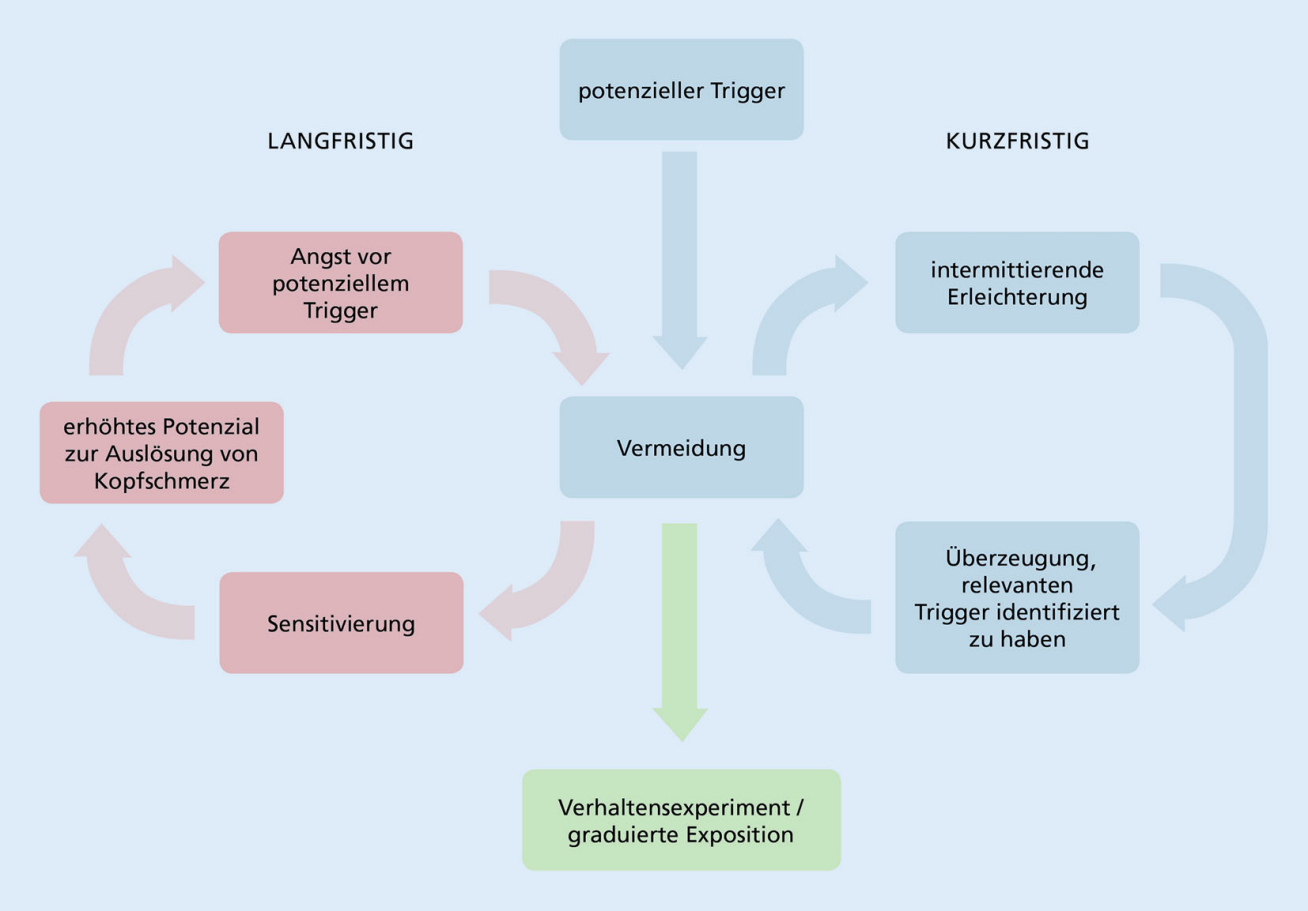

Die Entstehung der Krankheit ist multifaktoriell, wie bei anderen Schmerzerkrankungen auch kann ein biopsychosoziales Störungsmodell zugrunde gelegt werden [5, 26]. Bei den psychischen Faktoren, die den Krankheitsverlauf beeinflussen, spielt neben Stress die Emotion Angst eine wichtige Rolle. Angst ist in vielerlei Hinsicht mit Migräne assoziiert. So konnte gezeigt werden, dass Migränebetroffene im Vergleich zu Personen ohne Migräne mehr Ängste berichten [4, 17] und häufiger an Angststörungen leiden [9]. Eine höhere Kopfschmerzaktivität ist mit mehr Angstsymptomen assoziiert [4, 9]. Die Emotion Angst wiederum kann direkt zur Auslösung von Migräneattacken beitragen [28]. Außerdem kann Angst dazu führen, dass durchgeführte Behandlungen weniger wirksam sind [31]. Angst kann sich also ungünstig auf den Krankheits- und Behandlungsverlauf auswirken und somit zur Chronifizierung der Migräneerkrankung beitragen. Eine theoretische Grundlage hierfür bildet das Fear-avoidance-Modell (z. B. [38]), welches für primäre Kopfschmerzerkrankungen adaptiert wurde („trigger avoidance model of headaches“ [TAMH]; [25]; Abb. 1). Eine Erweiterung des Fear-avoidance-Modells stellt das Avoidance-endurance-Modell dar, welches zusätzlich zu angstassoziiertem Vermeidungsverhalten auch dysfunktionales Durchhalteverhalten integriert und für das Krankheitsbild Migräne anwendbar ist [33].

**Figure 2:**
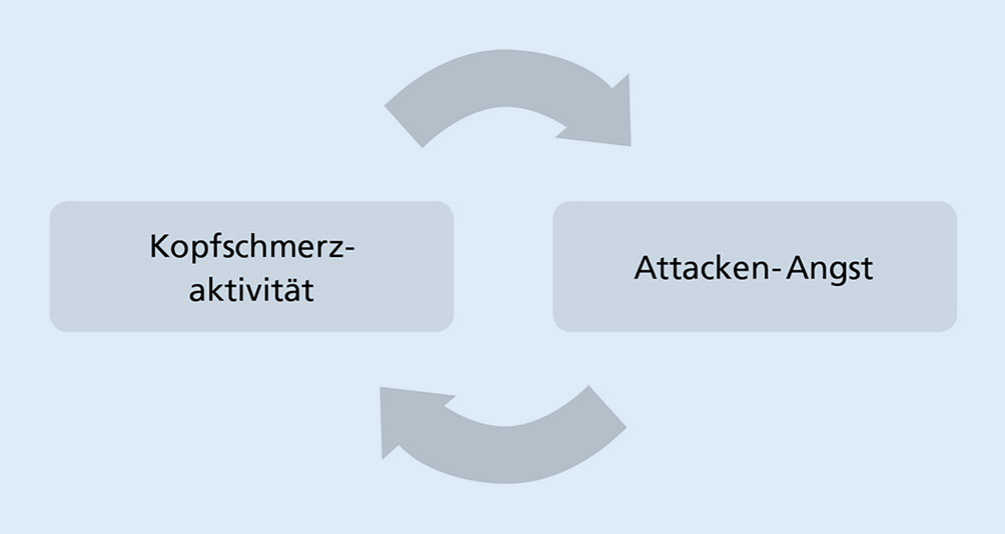

Neben einer erhöhten Komorbidität von klar definierten Angststörungen (z. B. Panikstörung [9]) und dem verstärkten Auftreten von allgemeinen Angstsymptomen [17] gibt es eine Reihe von sehr migränespezifischen Ängsten. Eine häufige Befürchtung bei Migränebetroffenen ist die antizipatorische Angst vor dem Auftreten einer Kopfschmerzattacke (sogenannte „Attackenangst“). In einer internationalen Studie mit Migränebetroffenen gaben 55 % der Befragten an, Angst vor einer auftretenden Kopfschmerzattacke zu haben [24]. Weitere spezifische Ängste im Kontext einer Migräneerkrankung können die Angst vor der Aura oder dem Erbrechen (Emetophobie) sein. Die Angst vor Kopfschmerzattacken kann bei Personen mit Migräne dazu führen, dass bereits im Vorfeld von möglichen Attacken („präventiv“) Schmerzmittel (Triptane, Analgetika) eingenommen werden. Durch eine zu häufige Präventiveinnahme kann sich ein langfristig übermäßiger Schmerzmittelkonsum etablieren, der zur Entstehung eines Kopfschmerzes durch Medikamentenübergebrauch führen und dadurch auch indirekt zur Erhöhung der Kopfschmerzaktivität beitragen kann [12, 29, 31]. Kopfschmerzaktivität und (Attacken‑)Angst können sich bei Migräne also wechselseitig „hochschaukeln“ (Abb. 2). Während der COVID-19-Pandemie kam es tendenziell zu einer Zunahme von Ängsten bei Migränebetroffenen (z. B. [13, 36]), wenngleich sich durchaus auch positive Effekte im Sinne einer reduzierten Kopfschmerzaktivität (z. B. durch geringere Stressbelastung, Abnahme von Reizdichte) beobachten ließen [10]. Angesichts der insgesamt hohen Prävalenz von Ängsten und deren ungünstigen Einflusses auf den Krankheitsverlauf ist es sinnvoll, Angstsymptome möglichst früh gezielt zu erfassen und zu behandeln.

## Diagnostik

### Entwicklung des Konzepts Attackenangst

Die Angst vor dem Auftreten von Kopfschmerzen bzw. einer Migräneattacke wurde erstmals im Jahr 2007 nosologisch betrachtet [29]. Dabei wurde Attackenangst als Subtypus einer spezifischen Phobie gemäß DSM-IV [1] konzeptualisiert und die Bezeichnung „Cephalalgiaphobie“ für das Phänomen ausgewählt. Als zentrales, angstassoziiertes Vermeidungsverhalten bei Cephalalgiaphobie wird ein Übergebrauch von Akutmedikamenten (v. a. Analgetika, Triptane) beschrieben. Das Konzept der Attackenangst als krankheitsspezifische Phobie wurde im Jahr 2013 von Giannini und Kollegen aufgegriffen [12]. Diese untersuchten eine Stichprobe von *N* = 126 Migränepatienten mit einem aus vier Items bestehenden strukturierten Interview, welches die Bildung eines „Cephalalgiaphobie-Scores“ ermöglicht. Drei der vier Items zielen dabei auf eine Erfassung des Konsums von Schmerzmedikamenten ab [12]. Eine höhere Attackenangst zeigte sich bei Migränepatienten mit einer hohen Attackenfrequenz sowie bei einem zusätzlichen Medikamentenübergebrauch. Die Ergebnisse bestätigten die Annahme, dass Attackenangst mit einer hohen Kopfschmerzaktivität sowie einem Medikamentenübergebrauch assoziiert sein kann. Ein Kritikpunkt ist die einseitige Fokussierung auf den Medikamentenübergebrauch als zentrales (Verhaltens‑)Kriterium der Attackenangst. Zwar gibt es Hinweise auf einen starken Zusammenhang zwischen Kopfschmerz durch Medikamentenübergebrauch (MOH) und Migräne (z. B. besteht im Vorfeld eines MOH in der Regel eine Migräneerkrankung; [37]). Allerdings ist der Anteil der Personen mit MOH unter den Migränebetroffenen insgesamt relativ gering. Einer aktuellen Querschnittsbefragung in Deutschland zufolge weisen nur ca. 1,9 % der Migränebetroffenen zusätzlich einen MOH auf [30], was zeigt, dass neben Medikamentenübergebrauch offensichtlich auch andere Aspekte eine Relevanz bei dem Phänomen Attackenangst haben.

### Fragebogen zur Attackenangst bei Migräne (FAMI)

Zur Erfassung des Konstrukts Attackenangst wurde im Jahr 2022 ein 29 Items umfassender Selbstbeurteilungsfragebogen (*Fragebogen zur Attackenangst bei Migräne* [FAMI]) entwickelt und an einer Stichprobe von *N* = 387 Migränebetroffenen validiert [19]. Die im FAMI enthaltenen 29 Aussagen (z. B. Item 10 „Ich befürchte, wegen einer Migräneattacke meinen Verpflichtungen nicht nachzukommen.“) werden jeweils auf einer fünfstufigen Likert-Skala (1 = starke Ablehnung bis 5 = starke Zustimmung) bewertet. Mittels Faktorenanalyse konnten drei gut interpretierbare Faktoren identifiziert werden: 1. Furcht vor negativen Konsequenzen, 2. Aufmerksamkeit und Antizipation, 3. Furchtvermeidung. Die Reliabilität (McDonalds ω) der Subskalen war gut (Subskala Aufmerksamkeit und Antizipation: ω = 0,88; Subskala Furchtvermeidung: ω = 0,85) bis exzellent (Subskala Furcht vor negativen Konsequenzen: ω = 0,91). Korrelationsanalysen lieferten Hinweise auf die konvergente Validität des FAMI. So korrelierten klinische Parameter (z. B. Kopfschmerztage) und korrespondierende Fragebögen (z. B. Pain Anxiety Symptoms Scale [22]) signifikant positiv mit allen Skalen des FAMI. Mit diesem Beitrag wird der FAMI erstmals in deutscher Sprache publiziert (Online-Zusatzmaterial). Bei der Auswertung und Interpretation des FAMI für Einzelpersonen ist zu beachten, dass noch keine Normstichprobe vorliegt (Infobox 1). Die vorhandenen Kennwerte der Studienstichprobe ermöglichen eine erste Orientierung bei der Interpretation von Werten (Tab. 1). Als Cut-off für klinisch relevante Attackenangst schlagen wir einen Gesamtsummenwert von ≥ 116 Punkten vor. Bei Personen, die allen 29 Items des FAMI zustimmen (was jeweils 4 Punkten entspricht), dürfte mit hoher Wahrscheinlichkeit eine klinisch relevante Angstsymptomatik vorliegen. Auch Personen, die nicht allen 29 Items zustimmen, bei denen aber eine starke Zustimmung (= 5 Punkte) durch niedrigere Werte auf anderen Items „kompensiert“ wird, würden mit unter diese Definition fallen.

**Table 1:** 

	Migräne mit/ohne Aura (*M* ± *SD*)	Chronische Migräne (*M* ± *SD*)	*t*^*a*^* (p)*
Gesamtskala (FAMI)	101,5 ± 20,3	108,7 ± 18,2	3,53 (< 0,001)
Skala 1: Furcht vor negativen Konsequenzen	33,3 ± 8,3	37,8 ± 6,4	6,00 (< 0,001)
Skala 2: Aufmerksamkeit und Antizipation	44,7 ± 9,4	45,8 ± 9,1	1,15 (0,252)
Skala 3: Furchtvermeidung	23,5 ± 5,8	25,1 ± 5,5	2,63 (0,009)

*M* Mittelwert, *SD* Standardabweichung

^a^Zweiseitiger *t*-Test für unabhängige Stichproben (α = 0,05)

Zusammengefasst ist der FAMI ein ökonomisches Messinstrument mit guten psychometrischen Eigenschaften, das in der klinischen Praxis und Forschung zur Erfassung von Attackenangst bei Personen mit Migräne angewendet werden kann. Faktorenanalytisch konnte gezeigt werden, dass das Konstrukt „Attackenangst“ neben kognitiven Aspekten (Furcht vor negativen Konsequenzen, Aufmerksamkeit) auch eine Verhaltenskomponente (Furchtvermeidung) umfasst. Das Medikamenteneinnahmeverhalten ist nicht in der Faktorenstruktur des FAMI enthalten. Der (übermäßige) Konsum von Akutmedikamenten stellt offensichtlich nicht das zentrale Vermeidungsverhalten bei Attackenangst dar. Andere angstmotivierte Verhaltensweisen wie das Vermeiden von Kopfschmerzauslösern (z. B. Absagen von sozialen Aktivitäten) sind möglicherweise relevanter. Dies steht in Einklang mit dem Befund, dass Medikamentenübergebrauch nur eine Minderheit von Personen mit Migräne betrifft und vor allem bei chronischer Migräne von Relevanz ist [30, 34].

### Kategoriale vs. dimensionale Diagnostik

Es stellt sich die Frage, ob die Konzeptualisierung von Attackenangst als spezifische Phobie sinnvoll ist. Die Angst, eine Migräneattacke zu erleiden und negative Konsequenzen zu erfahren, ist für Personen mit Migräne (insbesondere für Personen mit höherfrequenten Attacken) nicht immer unbegründet und kann je nach Inhalt der befürchteten Konsequenz durchaus angemessen sein. Dies erschwert die Anwendung der entsprechenden DSM-5- [2] bzw. ICD-10-Kriterien [8] für eine spezifische Phobie, da hier jeweils explizit eine Unverhältnismäßigkeit der Angst als diagnostisches Kriterium genannt ist. Während ein Kernmerkmal der spezifischen Phobien also die Irrationalität der jeweiligen Befürchtung (und eine Einsicht in die Übertriebenheit dieser) ist, können attackenassoziierte Ängste auch eine realistische Grundlage haben. Zwar können bestimmte Befürchtungen im Kontext von Migräneattacken tatsächlich unverhältnismäßig sein (z. B. Bezug auf die Toleranzfähigkeit von unangenehmen Empfindungen, wie beispielsweise „Ich werde die Schmerzen einer Migräneattacke nicht aushalten können“). Andere attackenassoziierte Ängste (z. B. zentraler Bezug auf potenzielle negative Reaktionen im sozialen Umfeld, wie beispielsweise „Ich werde negativ auffallen, wenn ich schon wieder bei der Arbeit fehle“) haben hingegen durchaus eine realistische Grundlage. So zeigt die klinische Erfahrung, dass Personen, die wiederholt migränebedingt am Arbeitsplatz ausfallen, tatsächlich negative Konsequenzen (bis hin zur Kündigung) erleben können.

Neuere nosologische Ansätze in der psychologischen Forschung postulieren zudem eine dimensionale Herangehensweise bei der Beschreibung von psychischen Störungen („HiTOP-Modell“; [21]). Mit dem FAMI steht ein dimensionales Messinstrument zur Verfügung, welches eine differenzierte Erfassung der Ausprägung von Attackenangst auf drei Subskalen ermöglicht. In der Versorgungspraxis sollte – wenn die Anwendung des FAMI nicht möglich ist – zumindest eine entsprechende Screeningfrage (z. B. „Machen Sie sich oft Sorgen, dass eine Migräneattacke auftreten könnte?“) gestellt werden, um dann ggf. eine vertiefte Exploration anzuschließen. Hier bieten sich Fragen nach Frequenz („Wie oft treten die Sorgen auf, wie lange halten diese an?“), Inhalt („Was sind die konkreten Befürchtungen?“) und Auswirkungen („Wie wirken sich die Sorgen aus?“) an.

## Behandlung

Sollte in der Anamnese deutlich werden, dass durch bestehende Attackenangst eine Beeinträchtigung der Lebensqualität bzw. ein relevanter Leidensdruck vorhanden ist, sind spezifische Interventionen indiziert.

### Verhaltenstherapeutische Interventionen

In der Behandlung von Ängsten und Angststörungen ist die kognitive Verhaltenstherapie die Methode der Wahl [3]. Es ist naheliegend, die im Kontext der verhaltenstherapeutischen Behandlung von Angstsymptomen bewährten Interventionen auch auf das Phänomen der Attackenangst anzuwenden. Eine differenzierte Darstellung von kognitiven Techniken zur Analyse und Bewältigung von Attackenangst bei Kopfschmerz findet sich bei Diezemann [7] sowie in dem Therapiemanual „Kognitiv-verhaltenstherapeutisches Migränemanagement, MIMA“ [18]. Nachfolgend werden essenzielle Bestandteile der kognitiv-verhaltenstherapeutischen Behandlung von migräneassoziierter Attackenangst vorgestellt (Infobox 2: Fallbeispiel).

#### 1. Akzeptanzbasiertes Vorgehen

Es sollte dem Patienten vermittelt werden, dass Attackenangst nicht mit dem Vorhandensein einer psychischen Störung gleichzusetzen ist. Bestehende Ängste sollten als nachvollziehbare Reaktion auf die Krankheits- und Lebensumstände des Patienten konnotiert werden. Durch die Entstigmatisierung und Entpathologisierung der Ängste kann eine erste emotionale Entlastung beim Patienten erfolgen. Außerdem ist die Botschaft wichtig, dass Attackenangst nicht vollständig „wegtherapiert“ werden kann. Durch eine realistische Zielsetzung (z. B. bessere Kontrolle über Intensität und Ausmaß der Angst anstelle von „Verschwinden der Angst“) kann unnötiger Druck beim Patienten (und beim Therapeuten) verhindert werden. Außerdem sollte berücksichtigt werden, dass Befürchtungen im Kontext von Attackenangst – im Gegensatz zu „klassischen“ Angststörungen – durchaus eine realistische Grundlage haben können. So können wiederholte Migräneattacken tatsächlich zu negativen Konsequenzen im privaten und beruflichen Bereich führen.

#### 2. Verhaltensanalyse der Attackenangst

**Figure 3:**
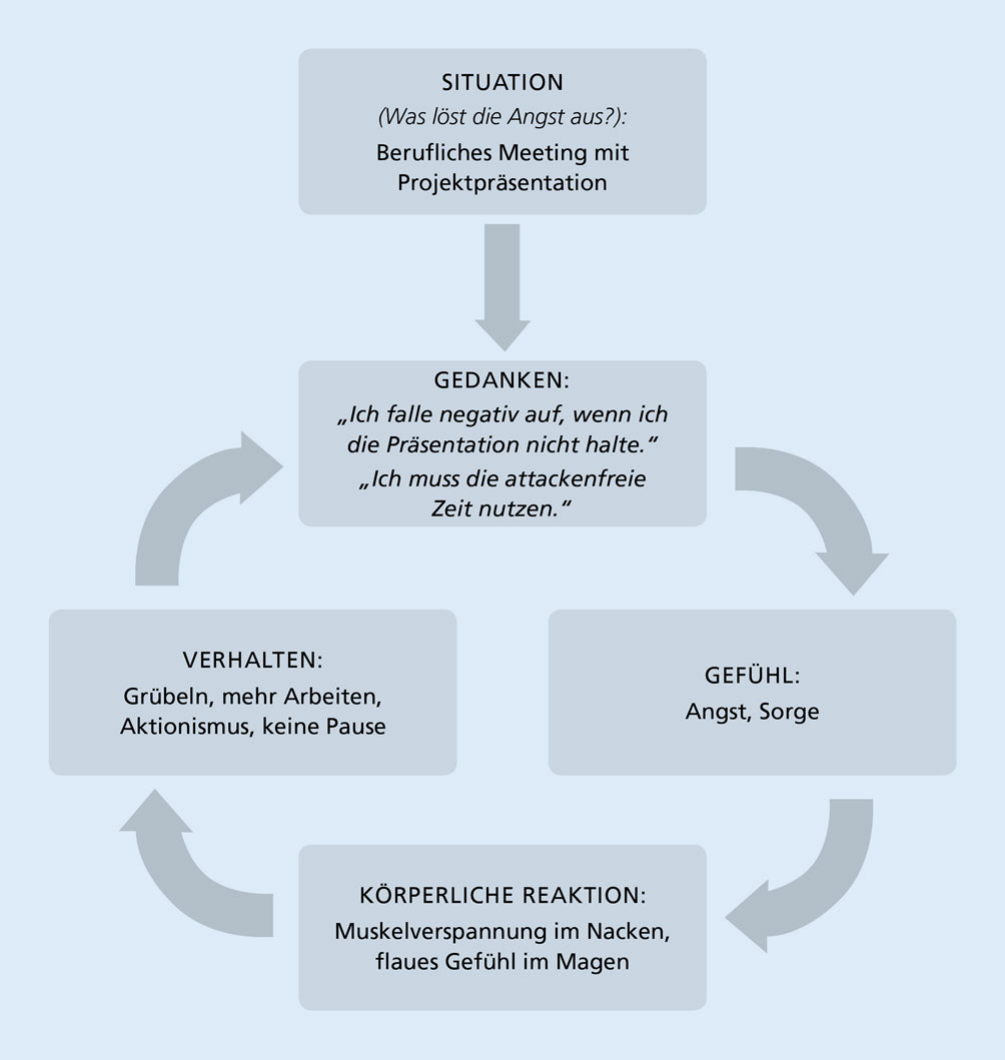

In Anlehnung an die gängigen Techniken der Verhaltensanalyse werden die Bedingungen für die Entstehung und Aufrechterhaltung der Attackenangst eruiert [16, 32]. Hierbei werden sowohl die Makroebene (z. B.: Welche Lebensereignisse haben die Attackenangst ausgelöst?) als auch die Mikroebene (z. B.: Was löst die Attackenangst unmittelbar aus? Welche Gedanken, Gefühle, Körperreaktionen und Verhaltensweisen sind mit der Attackenangst assoziiert?) berücksichtigt. Kritische Ereignisse für die Entstehung von Attackenangst können auf der Makroebene Schwellensituationen im Leben wie z. B. der Antritt einer neuen Arbeitsstelle sein. Nicht selten können von Patienten konkrete Veränderungen im beruflichen (z. B. neuer, kritischer Vorgesetzter, der für migränebedingte Ausfälle kein Verständnis hat) oder privaten Umfeld (z. B. neuer Lebenspartner, der wenig Verständnis für die Migräneerkrankung hat) retrospektiv als Auslöser von zunehmenden Attackenängsten berichtet werden. Für die Analyse des Verhaltens in konkreten Situationen bietet sich die Erstellung eines individuellen Teufelskreises der Attackenangst an (Abb. 3; [7, 18]). Im Vorfeld der Analyse von Attackenangst sollte eine entsprechende Psychoedukation erfolgen [18].

#### 3. Erarbeiten von Bewältigungsstrategien

Nach der individuellen Analyse der Attackenangst sollten konkrete Bewältigungsstrategien erarbeitet werden. Hier bieten sich (a) achtsamkeitsbasierte Techniken, (b) kognitive Interventionen sowie (c) expositionsbasierte Ansätze an.

- Unter Achtsamkeit versteht man eine Aufmerksamkeitslenkung auf die im aktuellen Moment vorhandenen Bewusstseinsinhalte, ohne diese zu bewerten. Achtsamkeit wird in formellen Übungen wie z. B. dem „bodyscan“ als auch in informellen Übungen im Alltag vermittelt und praktiziert. Ziele sind u. a. die *Schulung der Aufmerksamkeit* (durch die Steigerung der Konzentration auf das Hier und Jetzt wird das Lösen aus dysfunktionalen kognitiven Prozessen wie Grübeln verbessert) und das *frühzeitige Erkennen von ungünstigen Aufschaukelungsprozessen* (dadurch wird ein inneres Aussteigen aus emotionalen Belastungssituationen ermöglicht; [15, 27]).
- Bei bestehender Attackenangst können typische kognitive Verzerrungen wie z. B. die Tendenz zur Katastrophisierung oder zur Verallgemeinerung und der Einfluss dieser Gedanken auf die Emotion herausgearbeitet werden. Dadurch wird die Disputation bzw. das Hinterfragen dieser Gedanken angeregt und neue, günstigere Kognitionen (z. B. beruhigend, bewältigungsorientiert) können erarbeitet werden [7, 18]. Wenn das Hinterfragen der angstauslösenden Gedanken nicht gelingt, können Techniken der kognitiven Defusion angewendet werden [40]. Diese haben das Ziel der „Entschmelzung“ von den Gedanken, um sich von diesen zu distanzieren. Die kognitive Defusion führt häufig zu einer emotionalen Entlastung der Betroffenen und ermöglicht ein Erleben von Kontrolle.
- Manche Betroffene entwickeln durch die Attackenangst ein Vermeidungsverhalten von vermeintlichen Migränetriggern. Es kann dadurch zu einer Verschlechterung der Lebensqualität kommen (z. B. durch sozialen Rückzug, Aufgeben von Hobbies und Reisen). Hier bieten sich Verhaltensexperimente an, damit die Erfahrung gemacht werden kann, dass eine Attacke bei Konfrontation mit dem gefürchteten Trigger eventuell gar nicht eintritt. Dysfunktionale Kognitionen (hier: Verallgemeinerungen wie z. B. „Migräne tritt IMMER in vollen Räumen, bei lauter Musik, beim Reisen etc. auf“) können somit widerlegt werden. Bei übermäßigem Vermeiden (z. B. Verzicht auf Bildschirmarbeit) kann es zur Sensitivierung kommen, was zur Chronifizierung im Sinne des TAMH beitragen kann (Abb. 1). Alternativ zu Verhaltensexperimenten kann eine graduierte Exposition durchgeführt werden. Hierbei wird der Betroffene nach einem strukturierten Plan mit dem vermiedenen Reiz in zunehmenden Dosen konfrontiert, um die psychophysische Belastbarkeit wieder zu steigern [18].

### Medikamentöse Maßnahmen

Auch medikamentöse Interventionen können zu einer Reduktion von Attackenangst beitragen. Stellen erhöhte Anspannung und Angst wesentliche Anteile an der mit der Migräne einhergehenden Belastung dar, dann muss dies bei der Auswahl der medikamentösen Prophylaktika bzw. der medikamentösen Therapie von psychischen Komorbiditäten berücksichtigt werden. So wirken sich Betablocker häufig günstig auf Angstsymptome aus, möglicherweise, weil sie in der Lage sind, eine sympathische Überaktivität zu reduzieren. Gleichwohl ist die Studienlage in der Indikation „Angst“ zu Betablockern noch unbefriedigend [35]. Angstreduzierende Effekte und eine migräneprophylaktische Wirkung lassen sich auch mit trizyklischen Antidepressiva erreichen, von denen Amitriptylin zur Migräneprophylaxe zugelassen ist. Ebenfalls günstig auf eine Angststörung wirkt sich Opipramol aus, das als Therapie der zweiten Wahl sowohl in der Leitlinie zur Migräneprophylaxe als auch in der Leitlinie zur Behandlung von Angststörungen (hier: generalisierte Angststörung) als Option genannt wird [3, 6].

### Empirische Befunde zur Wirksamkeit

Es gibt nur wenige Studien, in denen die Wirksamkeit von verhaltenstherapeutischen Interventionen zur Reduktion von Attackenangst überprüft wurde. In einer multizentrischen Studie (*N* = 182 Migränebetroffene) von Fritsche und Kollegen (2010) wurde ein kognitiv-verhaltenstherapeutisches Kurzzeitprogramm mit einer Bibliotherapie verglichen [11]. In beiden Behandlungsgruppen wurden unter anderem Techniken im Umgang mit Attackenangst vermittelt. Die Teilnehmer beider Gruppen profitierten gleichermaßen mit einer signifikanten Reduktion der Kopfschmerzaktivität und der Medikamenteneinnahmetage. Die Effektivität des Therapieprogramms MIMA wurde in einer randomisiert-kontrollierten Studie (Klan und Kollegen 2022) mit insgesamt *N* = 106 Migränebetroffenen untersucht [20]. Dabei zeigte sich im Follow-up (12 Monate nach Therapieende) sowohl für das MIMA als auch in der aktiven Kontrollgruppe (Entspannungstraining, RLX) ein signifikanter Within-group-Effekt (Reduktion der Kopfschmerztage und der emotionalen Belastung). Ein Unterschied zwischen den beiden Interventionen (hier: MIMA vs. RLX) konnte nicht festgestellt werden. Kognitiv-verhaltenstherapeutische Interventionen, die spezifische Elemente zur Bewältigung von Attackenangst beinhalten, sind also potenziell wirksam. Eine Limitation der genannten Studien stellt der Mangel eines spezifischen Instruments zur Erfassung von Attackenangst dar. Insgesamt gesehen steht die Erforschung der Wirksamkeit von Interventionen zur Reduktion von Attackenangst bei Migräne erst am Anfang. Für zukünftige Studien zur Wirksamkeit verhaltenstherapeutischer Interventionen bei Migräne sollte Attackenangst mit dem FAMI gezielt erfasst werden. Auch im Kontext der Erforschung von medikamentöser Migräneprophylaxe wäre die Erfassung von Attackenangst als zusätzliches Outcome wünschenswert.

#### Infobox 1 Auswertung und Interpretation des Fragebogen zur Attackenangst bei Migräne (FAMI)

1. Berechnung der Skalensummen

Für jedes Item werden folgende Punktwerte vergeben: starke Ablehnung = 1 Punkt, Ablehnung = 2 Punkte, neutral = 3 Punkte, Zustimmung = 4 Punkte, starke Zustimmung = 5 Punkte. Alle Items sind in die gleiche Richtung gepolt (höhere Werte spiegeln höhere Angst bzw. stärkeres Vermeidungsverhalten wider). Der Skalensummenwert wird jeweils durch Addition der Punktwerte ermittelt:

- Skala 1 (Furcht vor negativen Konsequenzen): Item 5, 9, 10, 11, 12, 13, 14, 15, 16
- Skala 2 (Aufmerksamkeit und Antizipation): Item 1, 2, 3, 4, 6, 7, 8, 17, 19, 26, 27, 28, 29
- Skala 3 (Furchtvermeidung): Item 18, 20, 21, 22, 23, 24, 25

Außerdem kann ein Gesamtskalenwert (Addition aller Items) gebildet werden.

2. Interpretation

Zur Interpretation von Fragebogenwerten einzelner Personen kann ein Abgleich mit den vorhandenen Stichprobenkennwerten (Tab. 1) vorgenommen werden. Diese Stichprobe ist allerdings nicht repräsentativ. Es ist ein Vergleich mit der jeweiligen Subgruppe (Migräne mit/ohne Aura oder chronische Migräne) möglich. Bei einem Gesamtskalenwert von ≥ 116 Punkten liegt mit sehr hoher Wahrscheinlichkeit eine klinisch relevante Angstsymptomatik vor. Aber auch Skalenwerte, die im „Durchschnittsbereich“ liegen, können auf behandlungsrelevante Attackenangst hinweisen.

#### Infobox 2 Fallbeispiel

Frau F., 27 Jahre alt, hat ein erfolgreiches Studium der Wirtschaftswissenschaften absolviert. Sie leide seit ihrer Jugend an Migräne. Seit dem Abitur habe sich die Attackenfrequenz auf bis zu vier Attacken pro Monat erhöht. Eine Migräneattacke dauere ungefähr einen Tag, die Schmerzintensität sei meistens stark (ca. 7 auf der NRS). Als Akutmedikation nehme sie Zolmitriptan („hilft mir gegen die Schmerzen, aber meine Leistungsfähigkeit ist in der Attacke trotzdem beeinträchtigt“). Eine medikamentöse Prophylaxe sei bislang noch nicht eingeleitet, sie wolle „auch nicht mehr Medikamente einnehmen als nötig“.

Seit einem Jahr arbeite sie als Projektmanagerin. Ungefähr einmal pro Monat finde ein Meeting statt, in dem sie ihren Vorgesetzten den aktuellen Stand des Projekts vorstelle. Oft mache Sie sich bereits mehrere Tage im Vorfeld des Meetings Sorgen, attackenbedingt auszufallen: „Meistens geht es schon drei bis vier Tage vor dem Meeting los. Ich grüble dann darüber nach, dass ich negativ auffallen werde, wenn ich beim Meeting nicht dabei bin. Meine Anspannung steigt, ich merke das oft mit einer Verspannung der Nackenmuskulatur. Meistens schlafe ich die letzten Nächte vor dem Meeting schlecht. Am Morgen, wenn das Meeting stattfindet, ist dann die Attacke da.“ Auf weiteres Nachfragen berichtet die Patientin, dass sie in der Woche vor dem Meeting besonders viel und intensiv arbeite, schließlich müsse sie „die attackenfreie Zeit nutzen, um eine gute Präsentation zu erstellen“. Die empfohlenen Pausen bzw. Ruhephasen lege sie im Vorfeld des Meetings meistens nicht mehr ein. In der Behandlung wurde mit Frau F. zunächst eine individuelle Verhaltensanalyse durchgeführt (Abb. 3). Es wurde erarbeitet, dass die Sorge, durch einen attackenbedingten Ausfall negativ am Arbeitsplatz aufzufallen, durchaus eine realistische Grundlage hat. Die Annahme der Patientin, dass sie „die attackenfreie Zeit nutzen“ müsse, indem sie die letzten Tage vor dem Meeting ohne Pausen an ihrer Präsentation durcharbeite, wurde hingegen als dysfunktional klassifiziert. Hier kristallisierte sich ein überhöhter Leistungsanspruch („die Präsentation muss perfekt sein“) heraus. Das Verhalten der Patientin, in den Tagen vor der Präsentation auf die sonst üblichen Lebensstilmaßnahmen zur Attackenprophylaxe (z. B. Durchführung von Entspannungsübungen) zu verzichten, wurde als wenig zielführend bewertet. Mit der Patientin wurde erarbeitet, zukünftig im Vorfeld der Meetings besonders auf eine adäquate Work-Life-Balance zu achten und wiederholt Ruhephasen einzulegen. Tatsächlich konnte die Patientin durch diese Maßnahmen die Häufigkeit von attackenbedingten Ausfällen reduzieren, was sich auch positiv auf das Selbstwirksamkeitserleben der Patientin auswirkte („Ich habe jetzt nicht mehr so viel Attackenangst im Vorfeld von Meetings, weil ich jetzt weiß, was ich tun kann.“). Im Zusammenhang mit dem reduzierten Angstniveau sowie aufgrund der konsequenteren Anwendung nichtmedikamentöser Maßnahmen habe sich die Migräneaktivität auf nur noch bis zu zwei Attacken pro Monat reduziert.

## Fazit für die Praxis

- Attackenangst ist eine häufige Begleiterscheinung bei Migräne. Diese stellt eine zusätzliche emotionale Belastung dar und kann sich ungünstig auf den Krankheitsverlauf auswirken.
- Attackenangst hat kognitive Komponenten (Furcht vor negativen Konsequenzen sowie Aufmerksamkeit und Antizipation) und eine Verhaltenskomponente (v. a. Vermeiden von potenziellen Kopfschmerzauslösern).
- Zur Erfassung von Attackenangst liegt mit dem Fragebogen zur Attackenangst bei Migräne (FAMI) ein ökonomischer Selbstbeurteilungsfragebogen mit guten psychometrischen Eigenschaften vor.
- Im Rahmen der Kopfschmerzanamnese sollte potenzielle Attackenangst zumindest mit einer Screeningfrage adressiert werden.
- Zur gezielten Behandlung von Attackenangst liegen verhaltenstherapeutische und medikamentöse Interventionen vor. Ein Nachweis der Wirksamkeit dieser Interventionen in der Reduktion von migräneassoziierter Attackenangst steht noch aus.

## Supplementary Information




