# Supplementary material for: Fear of migraine attacks: diagnosis and treatment
Source: Schmerz. 2023 Apr 18;39(3):177–84. [Article in German] doi: 10.1007/s00482-023-00711-y (PMC10112828; doi:10.1007/s00482-023-00711-y)
Supplement: Supplementary file 1 — Fragebogen zur Attackenangst bei Migräne (FAMI) [file 482_2023_711_MOESM1_ESM.pdf]

Fragebogen zur Attackenangst bei Migräne (FAMI)

Als „Attackenangst“ wird die Sorge oder Angst vor dem Auftreten einer Migräneattacke bezeichnet. Vor allem im Vorfeld von wichtigen privaten oder beruflichen Ereignissen kann es zum Auftreten von Attackenangst kommen. Die Angst, durch eine Migräneattacke auszufallen, bedeutet oft eine zusätzliche emotionale Belastung und kann das Auftreten einer Migräneattacke begünstigen.

Der vorliegende Fragebogen wurde dazu entwickelt, das Ausmaß dieser Sorgen und Ängste zu erfassen. Nachfolgend finden Sie Aussagen, die sich auf mögliche Befürchtungen hinsichtlich des Auftretens einer Migräneattacke beziehen. Bitte geben Sie an, inwieweit Sie diesen Aussagen zustimmen. **Bitte beantworten Sie alle Fragen.** Sollte eine Frage gar nicht auf Sie zutreffen, kreuzen Sie „Starke Ablehnung“ an. Beziehen Sie sich bei Ihren Antworten auf Ihre gegenwärtige Lebenssituation.

Starke Ablehnung: Sie stimmen der Aussage auf keinen Fall zu oder Sie halten diese für völlig unzutreffend

Ablehnung: Sie stimmen der Aussage eher nicht zu oder Sie halten diese für unzutreffend

Neutral: Die Aussage ist weder richtig noch falsch, also weder zutreffend noch unzutreffend

Zustimmung: Sie stimmen der Aussage eher zu oder Sie halten diese für zutreffend

Starke Zustimmung: Sie stimmen der Aussage auf jeden Fall zu oder Sie halten diese für völlig zutreffend

---

Klan, T., Diezemann-Prößdorf, A., Guth, A.-L., Gaul, C. & Witthöft, M. Attackenangst bei Migräne: Diagnostik und Behandlung. *Schmerz* (2023). https://doi.org/10.1007/s00482-023-00711-y

	Starke Ablehnung	Ablehnung	Neutral	Zustimmung	Starke Zustimmung
1. In schmerzfreien Zeiten befürchte ich, dass bald eine Migräneattacke auftreten könnte.	☐	☐	☐	☐	☐
2. Ich habe Angst vor der nächsten Migräneattacke.	☐	☐	☐	☐	☐
3. In schmerzfreien Zeiten erinnere ich mich deutlich an die Belastungen der letzten Migräneattacke zurück.	☐	☐	☐	☐	☐
4. Ich versuche, das Eintreten einer Migräneattacke vorherzusagen (z.B. anhand von Regelmäßigkeiten in den Abständen der Migräneattacken).	☐	☐	☐	☐	☐
5. Vor wichtigen Terminen grüble ich über die Einschränkungen durch vergangene Migräneattacken.	☐	☐	☐	☐	☐
6. Die Einschränkungen durch vergangene Migräneattacken sind mir auch in schmerzfreien Zeiten bewusst.	☐	☐	☐	☐	☐
7. Ich habe Angst vor den Schmerzen einer kommenden Migräneattacke.	☐	☐	☐	☐	☐
8. Ich befürchte, durch meine Migräneattacken gesundheitliche Spätfolgen zu erleiden.	☐	☐	☐	☐	☐
9. Vor wichtigen Ereignissen (z.B. beruflich, familiär, privat) befürchte ich, eine Migräneattacke zu bekommen.	☐	☐	☐	☐	☐
10. Ich befürchte, wegen einer Migräneattacke meinen Verpflichtungen nicht nachzukommen.	☐	☐	☐	☐	☐
11. Ich habe Angst, dass ich wegen meiner Migräneattacken meine sozialen Aufgaben nicht zufriedenstellend erfüllen kann.	☐	☐	☐	☐	☐
12. Ich befürchte, dass Leute schlecht über mich reden, wenn ich aufgrund einer Migräneattacke Termine absagen muss.	☐	☐	☐	☐	☐
13. Ich habe Angst, durch meine Migräneattacken schöne Dinge im Leben zu verpassen.	☐	☐	☐	☐	☐
14. Ich befürchte, dass mich meine Migräneattacken daran hindern, das Leben zu genießen.	☐	☐	☐	☐	☐

	Starke Ablehnung	Ablehnung	Neutral	Zustimmung	Starke Zustimmung
15. Ich mache mir Sorgen, dass sich meine Migräneattacken negativ auf wichtige Beziehungen (z.B. Partnerschaft, Freundschaften, Kollegen) auswirken.	☐	☐	☐	☐	☐
16. Ich befürchte, dass ich durch meine Migräneattacken meine Lebensziele (z.B. beruflich, familiär, Freizeitbereich) nicht verwirklichen kann.	☐	☐	☐	☐	☐
17. Wenn ich Hinweissymptome auf eine Migräneattacke wahrnehme (z. B. Lichtempfindlichkeit), nehme ich vorsichtshalber Attackenmedikation (Schmerzmittel, Triptan) ein.	☐	☐	☐	☐	☐
18. Ich befürchte bei bestimmten äußeren Einflüssen (z.B. Wetter, Lichtreize, Menschenmengen, Lärm) eine Migräneattacke zu bekommen.	☐	☐	☐	☐	☐
19. Ich befürchte bei bestimmten inneren Zuständen (z.B. starke Emotionen, Stressbelastung, Müdigkeit) eine Migräneattacke zu bekommen.	☐	☐	☐	☐	☐
20. Ich befürchte, durch bestimmte Verhaltensweisen (z.B. Überanstrengung, unregelmäßiger Schlaf, Verzehr bestimmter Lebensmittel) eine Migräneattacke zu bekommen.	☐	☐	☐	☐	☐
21. Ich versuche bestimmte äußere Einflüsse (z.B. Lichtreize, Menschenmengen, Lärm) zu vermeiden, um keine Migräneattacke zu provozieren.	☐	☐	☐	☐	☐
22. Ich vermeide gewisse Verhaltensweisen (z.B. Überanstrengung, unregelmäßiger Schlaf, Verzehr bestimmter Lebensmittel), um keine Migräneattacke zu provozieren.	☐	☐	☐	☐	☐
23. Ich vermeide bestimmte innere Zustände (z.B. starke Emotionen, Stressbelastung, Müdigkeit), um keine Migräneattacke zu provozieren.	☐	☐	☐	☐	☐
24. Ich schirme mich von bestimmten äußeren Reizen ab (z.B. durch Kopfhörer, Schal, Sonnenbrille), um keine Migräneattacke zu provozieren.	☐	☐	☐	☐	☐

	Starke Ablehnung	Ablehnung	Neutral	Zustimmung	Starke Zustimmung
25. Ich sage Termine im Vorfeld ab, aus Sorge durch diese eine Migräneattacke zu provozieren.	☐	☐	☐	☐	☐
26. Nach dem Auftreten bestimmter innerer Zustände (z.B. starke Emotionen, Stressbelastung, Müdigkeit) achte ich besonders auf Anzeichen einer Migräneattacke.	☐	☐	☐	☐	☐
27. Ich horche regelmäßig in mich hinein, um Anzeichen für eine Migräneattacke zu erkennen.	☐	☐	☐	☐	☐
28. Ich achte besonders auf erste Anzeichen einer Migräneattacke, um möglichst früh reagieren zu können.	☐	☐	☐	☐	☐
29. Meine Angst vor einer Migräneattacke spüre ich mit körperlichen Symptomen (z.B. Mundtrockenheit, Herzklopfen, innere Unruhe).	☐	☐	☐	☐	☐
